# Effect of Laryngeal Squamous Cell Carcinoma Tissue Implantation on the Chick Embryo Chorioallantoic Membrane: Morphometric Measurements and Vascularity

**DOI:** 10.1155/2015/629754

**Published:** 2015-10-11

**Authors:** Virgilijus Uloza, Alina Kuzminienė, Sonata Šalomskaitė-Davalgienė, Jolita Palubinskienė, Ingrida Balnytė, Ingrida Ulozienė, Viktoras Šaferis, Angelija Valančiūtė

**Affiliations:** ^1^Department of Otorhinolaryngology, Lithuanian University of Health Sciences, LT-50009 Kaunas, Lithuania; ^2^Department of Histology and Embryology, Lithuanian University of Health Sciences, LT-50009 Kaunas, Lithuania; ^3^Department of Physics, Mathematics and Biophysics, Lithuanian University of Health Sciences, LT-50009 Kaunas, Lithuania

## Abstract

*Background*. The aim of this study was to develop chick embryo chorioallantoic membrane (CAM) model of laryngeal squamous cell carcinoma (LSCC) and to evaluate the morphological and morphometric characteristics and angiogenic features of it. *Methods*. Fresh LSCC tissue samples obtained from 6 patients were implanted onto 15 chick embryo CAMs. Morphological, morphometric, and angiogenic changes in the CAM and chorionic epithelium were evaluated up to 4 days after the tumor implantation. Immunohistochemical analysis (34*β*E12, CD31, and Ki67 staining) was performed to detect cytokeratins and tumor endothelial cells and to evaluate the proliferative capacity of the tumor before and after implantation on the CAM. *Results*. The implanted LSCC tissue samples survived on the CAM in all the experiments and retained the essential morphologic characteristics and proliferative capacity of the original tumor. Implants induced thickening of both the CAM (103–417%, *p* = 0.0001) and the chorionic epithelium (70–140%, *p* = 0.0001) and increase in number of blood vessels (75–148%, *p* = 0.0001) in the CAM. *Conclusions*. This study clarifies that chick embryo CAM is a relevant assay for implanting LSCC tissue and provides the first morphological and morphometric characterization of the LSCC CAM model that opens new perspectives to study this disease.

## 1. Introduction

Laryngeal squamous cell carcinoma (LSCC) is one of the most common malignant tumors of the respiratory tract with an estimated incidence rate of 10/100 000 cases in males in Europe [1]. The overall 5-year survival of patients with this carcinoma localization in Europe was 63% in the period of 1995 to 2003. Despite the up-to-date treatment using advanced chemoradiation therapy and modern surgical techniques, the survival rate is not increasing remarkably within the last 30 years [1].

A better understanding of the biological mechanisms that control progression of LSCC would provide new and more successful strategies for tumor management. However, current* in vivo* models for human LSCC investigation do not simulate enough tumorigenic phenotypes of cancer and data about experimental evaluation of carcinogenesis, angiogenesis, and metastatic potential of LSCC in a live medium are insufficient [2, 3]. Therefore, we suggest establishing chick embryo chorioallantoic membrane (CAM) assay for this type of tumor as a medium that reveals numerous unique properties and advantages [4, 5].

Because the chick embryo CAM model has been used for scientific purposes for decades, the system is quite well described in the literature and some evident benefits of the CAM assay are emphasized [6, 7]. The immatureness of the chick embryo immune system allows using different cell types and cells from different tissues and species. Consequently, the chick embryo CAM assay is considered as simple, rapid, and cost effective, if compared with most* in vivo* models [4].

Therefore, we propose that the chick embryo CAM model can be successfully used to characterize LSCC morphology, invasion, angiogenic response* in vivo*, and metastatic properties. Remarkable proof is the evidence that the chick embryo CAM model has substantially contributed to several Nobel Prize laureates' scientific discoveries including the first known oncogene [8], neural growth factor based on effects of mouse tumor transplantation [9], and the interaction between tumor viruses and the genetic material of the cell [10].

The chick embryo develops 21 days until hatching out. The CAM is formed on days 4 to 5 of incubation as a consequence of fusion of mesodermal layers of outgrowing allantois and the chorion. The newly formed membrane is composed of chorionic and endodermal epithelia with an intricate plexus of arteries, veins, and capillaries. The highly vascularized nature of this assay is a considerable advantage which greatly stimulates the growth of the xenogeneic tumor and facilitates the analysis of angiogenic effect of implanted tumor on the CAM [11]. This feature determines the use of the CAM as a perfect medium for tumor implanting, studying carcinoma behavior and development. It is probably the most widely used* in vivo* assay for studying angiogenesis [4, 11, 12].

One of the greatest features of chick embryo CAM assay is an incomplete lymphoid system which is not fully developed until the late stages of incubation. The chick embryo may serve as a naturally immunodeficient host that is efficient for maintaining implanted tumor tissues without species-specific restrictions [11]. It is known that after the chick embryo becomes immunocompetent (the 18th day of incubation), both acute and chronic inflammatory responses of the CAM to biomaterials become similar to those of mammalian ones [4]. Data of the literature show that chick genes have a single human orthologue with an accuracy of about 60%. Chick and human orthologous genes reveal lower sequence conservation (75.3%) than rodent and human do (88%) [4]. Therefore, the avian model can be used for research in more fields of investigation compared to rodent ones. Moreover, no special permission from the Animal Rights Protection Committee is needed to perform the experimentation with chick embryo in both the European Union and USA. A great support to perform this type of investigation is approbation obtained from the US Food and Drug Administration and the Communication Department of the European Commission (2006) for the products that are preclinically evaluated using this assay [4].

Most of the avian experimental models such as human osteosarcoma, human colon carcinoma, and others used cells from the tumor cell lines implanted in the chick embryo CAM [11, 13, 14]. However, it can be presumed that this type of experiment while implanting cultivated tumor cells loses most of the natural physiological and histological features of the original tumor. Several* in vitro* models have been developed in the last few decades to investigate the oncogenic phenotypes of different malignant tumors. However, most of these models employed monolayer cell cultures, making these assays difficult to translate to clinical applications [6].

Following the experience obtained from glioblastoma tumor implantation on the chick embryo CAM [15], we suggested implanting fresh laryngeal tumor samples onto the chick embryo CAM [16], expecting that the tumor will retain its physiological properties and will show analogous behavior as in its natural environment. We demonstrated that fresh LSCC tissue samples remain viable with their main histological features up to 4 days after implantation onto the chick embryo CAM.

The aim of this study was to evaluate the morphological and morphometric characteristics and angiogenic features of the chick embryo CAM LSCC model. In this study, we used the chick embryo CAM for the first time to investigate the angiogenic effect of LSCC and to provide the morphometric characteristics of the CAM LSCC model. The implanted tumor induced considerable morphological changes of the CAM structures and demonstrated significant instigated vascularization of the host membrane.

## 2. Materials and Methods

### 2.1. Incubation and Egg Opening

Fertilized hen eggs (*Cobb-500*) were obtained from the local hatchery (Dovainonių Paukštynas, Lithuania) and were incubated at 37.7°C temperature and 59-60% relative humidity with permanent ventilation and rotation. On the third embryonic day (approximately 72 hours of incubation) eggs' shells were sterilized with 70% ethanol solution. The blunt part of the egg was punctured searching for the air chamber. Two milliliters of albumen was removed in order to set down the developing embryo. Then, an oval window of about 1.0 cm² on the top of the shell of each egg was opened using a high speed drill. All embryos were examined for possible malformations or signs of local bleeding. Those embryos that did not satisfy the study requirements were discarded.

In order to prevent embryos from dehydration and to capacitate the continuity of the experiment the shell windows were covered with transparent sterile tape. After this procedure, the prepared eggs were placed back into the incubator and kept under the same conditions without rotation for 4 to 6 days until implantation of the LSCC tissue.

### 2.2. LSCC Tissue Samples

Fresh LSCC tissue samples (*N* = 6) of at least about 0.5 × 1.0 × 0.5 cm in size were obtained from 6 patients at the Department of Otorhinolaryngology during laryngeal surgery. Diagnosis of the LSCC was proved at the Department of Pathology. The LSCC tissue samples were transported to the laboratory of the Department of Histology and Embryology in isotonic saline solution at ambient temperature (18–20°C) and then implanted onto the chick embryo CAM within 45–60 minutes.

Investigations in the present study were performed in accordance with the principles outlined in the Declaration of Helsinki and approved by Kaunas Regional Bioethics Committee (P1-BE-2-34/2007). Histologically confirmed LSCC tissue samples were acquired in accordance with the protocol approved by the Institutional Review Board of LUHS. Written Informed Consent was obtained from the patients before surgery and patients' identifiers were removed to ensure anonymity.

### 2.3. LSCC Tissue Implantation onto the CAM

LSCC implantation was performed on the 7th, 8th, or 9th day of eggs' incubation when the CAM is already formed. Each LSCC tissue sample obtained directly from the operating room was sliced into approximately 8 mm³ pieces. Each piece of the tumor (1 piece per egg) was gently placed on the outer surface of the CAM near the biggest apparent vessel of the membrane, that is, using classical technique as it is described by Cushman et al. [17]. On the 11th day of eggs' incubation, that is, after 48, 72, and 96 hours of tumor implantation, two eggs were reopened and live-fixed in the 10% formalin solution. CAMs with the adhering tumors were excised and fixed in formalin for 5 days. Eight control CAMs were obtained on the 11th day from eggs that were incubated and proceeded under the same protocol, except the LSCC tissue implantation.

### 2.4. Tissue Sampling and Histology

Formalin-fixed and paraffin-embedded samples of approximately 0.5 × 2.5 cm in size from each CAM with LSCC implant were sliced into 3 *μ*m thick sections and stained with hematoxylin and eosin (H&E) for histological and morphometric evaluation. Histological evaluation of the samples was performed with the cold light microscope OLYMPUS BX40F4 (Olympus Optical Co. Ltd., Japan) under 10x magnification using CellSens Dimension 1.9 Digital Imaging Software for Research Applications (Olympus Corporation of the Americas, USA).

### 2.5. Immunohistochemistry

For immunohistochemical examination, the 3 *μ*m thick slices of paraffin-embedded CAMs with LSCC implants as well as original tumor tissue slices were mounted on poly-L-lysine coated glass slides. After deparaffinization with xylene and rehydration the sections were pretreated with antigen-retrieval solution (0.01 mol/L of citrate buffer, pH 6) in a pressure-cooker and then incubated: (1) with cytokeratin monoclonal antibodies (clone 34*β*E12, dilution 1 : 50) for identification of high molecular weight cytokeratin (HMW CK), because previous studies have shown squamous cell carcinomas being positive for these antibodies [18], (2) with monoclonal mouse anti-human CD31 (endothelial cell clone JC70A, dilution 1 : 40) for detection of vascular endothelial cells in tumor tissue [19, 20], and (3) with monoclonal mouse anti-human antibody for Ki67 (clone MIB-1, dilution 1 : 50) to identify nuclei of proliferating tumor cells [21].

All antibodies were purchased from Dako A/S (Glostrup, Denmark). Antibodies' detection using commercially available kit EnVision Plus-HRP, Dako A/S, was performed following the protocols of the provider. Sections were counterstained in weak Mayer's hematoxylin, dehydrated, cleared, and mounted for the light microscopy.

### 2.6. Histochemistry of Mesodermal CAM Vessels

**Figure 1:**
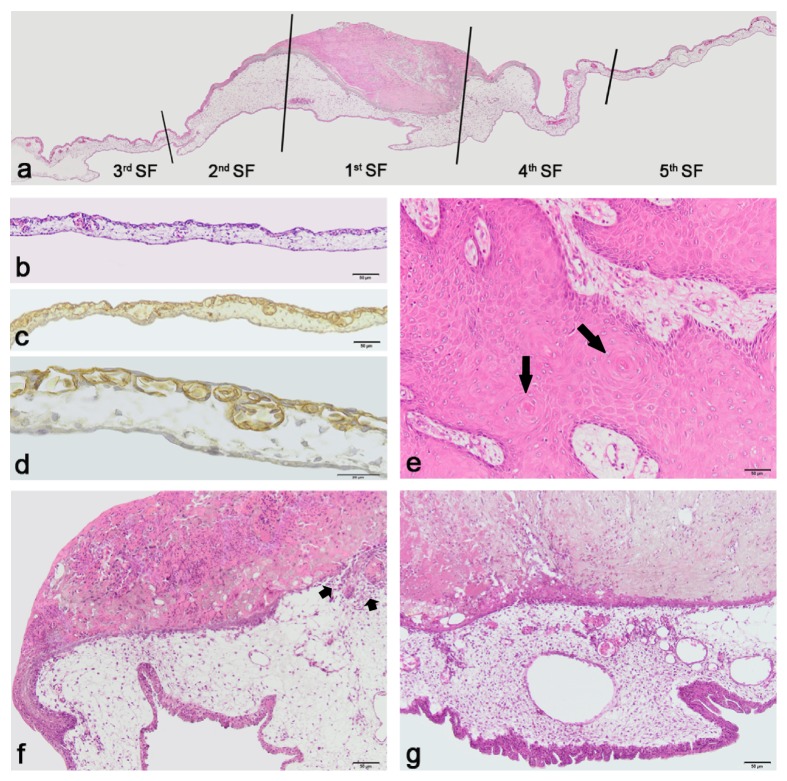
Histological appearance of the control and experimental CAM and LSCC tissue. (a) Sight fields of CAM with LSCC implant; original magnification 4x. (b–d) The control CAMs: stained in H&Е (b); CAM's blood vessels revealed with* Sambucus nigra* lectin (c, d). (e) Moderately differentiated squamous cell carcinoma of the larynx. Accumulation of atypical cells with concentrically arranged keratinized cells (“carcinoma pearls”: long arrows) was observed in the original LSCC tissue. (f) Thickened CAM and chorionic epithelium of experimental group with LSCC implant: invasion through epithelium (short arrows). (g) Increased vascularity under the LSCC implant. (b, c, e, f, and g) Bars 50 *μ*m; original magnification 10x; (d) bar 20 *μ*m; original magnification 40x.

For visualization of CAM blood vessels, paraffin-embedded tissue samples were sliced in 3 *μ*m thick slices and mounted on poly-L-lysine coated glass slides. Sections were rehydrated as previously described and pretreated with streptavidin/biotin blocking kit (Vector, USA). In order to highlight endothelium of blood vessels in chick embryo CAM, slices were stained with 10 *μ*g/mL biotinylated* Sambucus nigra* bark lectin (Vector, USA) [22]. The VECTASTAIN* Elite ABC* kit was used (Vector, USA) to detect biotinylated molecules. Enzyme activity sites were visualized using DAB chromogen solution (Dako, Denmark). Sections were counterstained in Mayer's hematoxylin, dehydrated, cleared, and mounted.* Sambucus nigra* lectin specifically binds to chick endothelium; therefore, the blood vessels were seen brown under the light microscope (Figures 1(c) and 1(d)).

### 2.7. Histomorphometric Analysis

Histomorphometric evaluation of the CAM parameters was performed on the images obtained with Olympus digital camera (Olympus U-CMAD3, Philippines). To perform accurate morphometric analysis each CAM section was divided into 5 sight fields (SFs) (Figure 1(a)). The central location directly under the implanted tumor was defined as the first SF, the 2nd and 4th SFs were defined as neighboring sites, and the 3rd and 5th SFs were defined as distant SFs, respectively. The thicknesses of both the CAM and the chorionic epithelium were measured in all SFs. The parameters measured only in central and neighboring SFs (the 1st, 2nd, and 4th) were as follows: (1) number of blood vessels with the smallest diameter of not less than 8 *μ*m per constant length of the CAM section and (2) mean area of the counted blood vessels' cross-section. The latter were identified following the endothelial cells and erythrocytes inside the lumen of blood vessels in* Sambucus nigra* lectin (Figures 1(c) and 1(d)) and H&E stained sections, according to the systematic sampling approach of Russ and Dehoff [23]. Eight control CAMs were processed under the same conditions, except that measurements of each parameter were performed in five random SFs (Figures 1(b)–1(d)).

### 2.8. Statistical Analysis

Statistical analysis was performed using IBM SPSS Statistics for Windows, Version 20.0 (Armonk, NY: IBM Corporation Software). Data were presented as the mean ± standard deviation (SD). Student's *t*-test was used to test hypothesis with respect to equality of means. The size of the differences among the mean values of the groups was evaluated by estimation of type I and type II errors (*α* and *β*) of the tests. The difference was considered to be significant if *β* ≤ 0.2 and *α* = 0.05. The correlations among the number of blood vessels and the thickness of the CAM and the thickness of epithelium of the CAM were evaluated using Pearson's correlation coefficient (*r*). The level of statistical significance for testing statistical hypothesis was 0.05.

## 3. Results

The laryngeal squamous cell carcinoma tissue samples (*N* = 6) were tested on chick embryo CAMs (*N* = 120). In this paper we evaluate the effect of LSCC for those CAMs that were cut off on the 11th incubation day, that is, 2, 3, and 4 days after tumor tissue implantation (*N* = 15). All 15 CAMs were evaluated morphologically in numerous sections and morphometric parameters were obtained in 5 SFs of 4 nonserial sections (Figure 1(a)). Three hundred SFs were measured accordingly.

CAMs (*N* = 8) without implants served as controls. Measurements of control CAMs were made in 160 SFs using the same methods (Figures 1(b)–1(d)).

The original LSCC tissue was evaluated histologically. Typical signs of the tumor were observed: the parenchyma consisted of atypical epithelial cells with irregular nuclei and increased number of nucleoli. Accumulation of atypical cells with concentrically arranged keratinized cells (“carcinoma pearls”) was observed (Figure 1(e)). The surrounding stroma was composed of loose connective tissue showing different level of infiltration by monomorphonuclear cells.

### 3.1. Histological and Immunohistochemical Characteristics of Implanted LSCC Tissue

The implanted LSCC tissue samples consisted of solid pieces of polymorphous atypical squamous epithelial cells with large irregular nuclei and increased mitosis, while observing one or several prominent nucleoli and abundant acidophilic cytoplasm. The tumor cells retained their vitality in 2, 3, and 4 days after implantation and the apparent influence of the LSCC on the CAM was observed (Figures 1(f) and 1(g)). The implanted tumor tissues on the chick embryo CAM in all the cases were visibly adhered to the host CAM and never flowed away.

**Figure 2:**
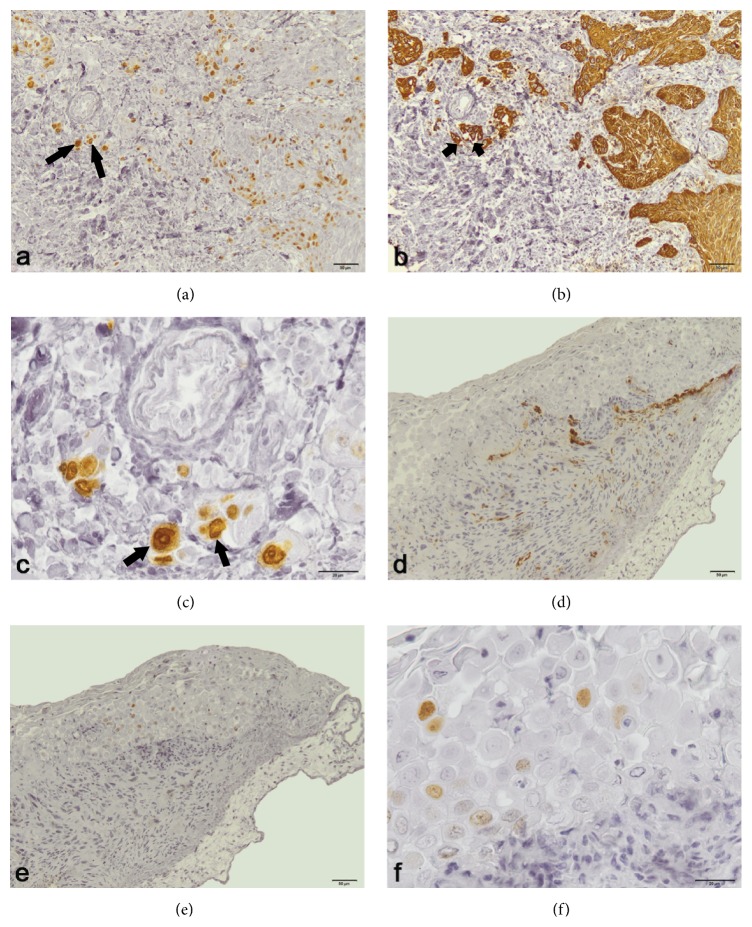
An immunohistochemical study of LSCC tissue before and after implantation on CAM. (a, c) The original tumor cells, which showed high positivity for the Ki67 (long arrows). (b) The original tumor cells (the same site as in (a)) were also positive for HMW CK (short arrows). (d) Endothelial cells of implanted tumor blood vessels were positive for CD31. (e, f) Ki67 positive nuclei of implanted LSCC cells. (a, b, d, and e) Bars 50 *μ*m; original magnification 10x; (c, f) bars 20 *μ*m; original magnification 40x.

The HMW CK (CK34*β*E12) was expressed in the cytoplasm of the original LSCC epithelial cells. Epithelial cells of the LSCC implanted on the CAM also showed high positivity for the HMW CK. Endothelial cells of the blood vessels were positive for CD31 in both the original and implanted LSCC tissues. Cellular marker for proliferation Ki67 was positive in the nuclei of the original tumor cells (Figures 2(a) and 2(c)) also showing positivity for HMW CK (Figure 2(b)). Expression of CD31 in implanted LSCC blood vessels (Figure 2(d)) and Ki67 in its epithelial cells (Figures 2(e) and 2(f)) indicated that the implanted tissue retained features of the original tumor and preserved proliferative capacity even after 96 hours after the implantation.

### 3.2. Morphometric and Morphologic Characteristics of the CAM

The morphological features of the CAMs' reaction induced by the LSCC implants were similar in all specimens of the experimental group. Thus, there were no significant differences between morphological features of the different CAMs with implants obtained from the same patient as well. Results of the morphometric analysis of the CAMs in experimental and control groups are presented in Table 1. The mean thickness of the CAM under the implanted LSCC in the experimental group was statistically significantly (*p* = 0.0001) increased in all 5 SFs comparing to that of the control group. The largest difference between the thickness of the experimental and the control CAMs was found in the central SF (1st SF) and reached 417%. However, in the distant SFs (3rd and 5th SFs) the difference was less and reached 103–109%, respectively. Furthermore, in the experimental group the host CAM in the central SF was statistically significantly thicker than in the neighboring and the distant SFs, respectively (*p* < 0.05).

Table 2 shows the mean thickness of the CAMs in both experimental and control groups 2, 3, and 4 days after the LSCC tumor implantation (i.e., totally 11 days of incubation). The mean thickness of the CAM was statistically significantly higher (*p* = 0.001) in the experimental group versus control group already 2 days after the tumor implantation reaching 177%. Of note, on the 4th day after the LSCC tumor implantation that difference reached 401%. There was no positivity for the HMW CK in the mesenchymal layer or endodermal epithelium of the CAM. Expression of CD31 and Ki67 was not detected in the CAM as well.

### 3.3. Morphometric and Morphologic Characteristics of the CAM Epithelium

The chorionic epithelium in the experimental group was found to be thickened in comparison with the control group (Table 3) and it appeared squamous and stratified, consisting of 5-6 layers. The mean thickness of the epithelium under the LSCC implant was statistically significantly (*p* = 0.0001) higher as compared with the control group. The highest difference was found in the 1st SF (140%) while in the distant SFs these differences reached 70 and 75%, respectively.

All implanted tumors induced similar morphometric characteristics of the CAM epithelium under the LSCC implants. No significant differences were found in the morphometric characteristics of the epithelium after implanting the same patient's tumor on different CAMs (*p* > 0.05). However, there were certain regions of the CAM under the LSCC implants with thinned and even discontinuous epithelium (Figure 1(f)) and signs of tumor cells' invasion. Adjacent mesenchyme showed a dense accumulation of blood vessels immediately below the implant (Figure 1(g)).

The keratogenic metaplasia in the chorionic epithelium just beneath the implanted LSCC was detected by positive immunostaining with HMW CK. However, keratogenic metaplasia has never been found in the distant SFs of the experimental CAMs, as well as in the CAM epithelium of the control group.

### 3.4. Histomorphological Characteristics of the Vascularity of the CAM

Histomorphometric evaluation revealed a statistically significant difference of the mean number of CAM blood vessels between the experimental and control groups (*p* = 0.0001) (Table 4). The experimental group had much higher mean number of blood vessels per constant length of the CAM, thus demonstrating measurable evidence of increased vascularity. The highest difference from the control CAM was in the 1st SF reaching 148%; however, in neighboring SFs the difference was less: 92% and 75%, respectively.

The mean number of blood vessels under the LSCC tissue implant (the 1st SF) was found to be significantly higher in comparison with the neighboring sites (*p* < 0.05) of the experimental CAM.

The statistically significant (*p* < 0.05) moderate positive correlations between the number of blood vessels and the thickness of the CAM (*r* = 0.65), as well as the thickness of epithelium of the CAM (*r* = 0.37), were revealed in the experimental group.

As shown in Table 5, the mean area of blood vessels' lumen in the experimental group was statistically significantly larger in all SFs if compared with that of the control group. In the 1st SF the difference between the experimental and control groups reached 155%, in the neighboring SFs, 106% and 82%, respectively.

## 4. Discussion

The chick embryo CAM model has long been used for the investigation of angiogenesis, oncogenesis, and tumor metastasis [24–26]. This model provides a naturally immunodeficient host that accepts implantation from various tissues and species and therefore can be used for xenoimplantation of different types of cells. The extraembryonic membranes that are connected to the embryo through a continuous extraembryonic vessel system are readily accessible for experimental manipulation and observations [27]. Despite the evident advantages of the CAM assay and its natural immunodeficient environment, the chick embryo CAM model is still relatively rarely used for implanting of human tumors. Nevertheless, there are several reports about the employment of the CAM assay as a reliable model to study various human tumors, namely, melanoma [28], prostatic cancer [29], glioblastoma [15, 30], human colon carcinoma [13], giant cell tumor of bone [14], sarcoma [31], and head and neck squamous cell carcinoma [6].

However, there are only sporadic reports in the literature about the use of the CAM assay for biological studies of human laryngeal tissue: for establishment of LSCC cell lines [2] and for CAM analysis of cellular laryngeal scaffolds showing induction of a strong* in vivo* angiogenic response [24].

On the other hand, most experiments with chick embryo CAM reported in the literature used tumor cell lines that did not represent the natural physiological features of a solid tumor. Experiments with cell lines might not fully reflect the wide heterogeneity of human malignancies, because of poor correlation between the behavior of single cell lines* in vitro* and tumors encountered in patients. Depurated cancer cell lines differ genetically from the original cancers in patients, because these cells have a phenotype adapted to culturing on plastic substrates that are commonly employed in xenograft experiments [31]. Positive performance of an exploratory drug in experimental xenografts of different human cancer cell lines is not predictive enough of compound efficacy in the clinical setting [32].

The use of fresh tumor samples for the CAM assay preserves the original tumor microenvironment of the heterogeneous tumor cell population and the associated matrix allowing natural interactions between the different cell populations in the sample [31]. Therefore, preservation of the microenvironment is a theoretical benefit of using fresh tumor samples.

The results of our study indicate that LSCC tissue samples outlived on the CAMs sustaining strongly adhered to the membranes in all the experiments despite the short term of interaction (2 to 4 days after implantation). All examined implants retained essential characteristics of the donor tumor specimens from living individuals with LSCC. It is important to note that all LSCC implants remained with their main histological features and no signs of necrosis were observed. Thus, the results of the present study show that the CAM assay can be used to analyze fresh material derived from LSCC. This is the first* in vivo* model for LSCC which opens new perspectives to study this disease and tumor aggressiveness and to assess tumor responses to new therapeutic agents.

We have noticed that LSCC tissues induced significant changes of all the structures of the host medium starting from the 2nd day after tumor implantation while having stayed on the CAM. The observed thickening of the mesenchyme with increased density of mesenchymal cells and thickening of chorionic epithelium in the CAM under the tumor implant can be explained as the result of action of the growth stimuli factors that are coming from the implanted LSCC tumor tissue and the nonspecific inflammatory reaction of the CAM due to the implant [33, 34].

Examination of CAM sections suggested that partial thickening in the mesenchyme between the outer and inner epithelium may be due to edema [35]. During the investigation with the uncoated dialysis capillary or by applying the Thermanox tissue culture cover slips onto the CAM, a high density of inflammatory cells, such as heterophils, and giant mast cells with associated fibrosis were found. The stroma of the CAM showed fibrocyte proliferation, leucocyte infiltration, and clusters of dispersed ectodermal epithelial cells [36–39]. As chick embryo CAM is accepted to be a naturally immunodeficient host [11] until day 18 of incubation [4], the inflammatory response of the chorioallantoic membrane to biomaterials is explained as the result of appearance of nonlymphoid avian leukocytes, mast cells, basophils, thrombocytes (functional analog to platelets), and monocytes that represent nonspecific inflammatory reaction [33].

Angiogenesis plays a critical role in many normal physiological processes, as well as in tumor neovascularization [34]. Establishment and growth of malignant tumors are critically dependent on their ability to stimulate the formation of new blood vessels from preexisting vasculature to support their metabolic needs [34, 40]. Thus, angiogenesis facilitates tumor growth and spread [6].

In head and neck tumors, increased angiogenesis has been associated with an unfavorable prognosis in many studies; however, prognostic relevance of angiogenic factors in laryngeal tumor development has been questioned [41–43]. More recent studies emphasized that increased LSCC tumor angiogenesis was an early event in laryngeal tumor development and positively correlated with local and locoregional relapse and lethal outcome of the disease [44, 45].

Chick embryo CAM being rich in developed arteries, veins, and capillary plexus also accompanied by evolved nutrition delivery system is accepted to be one of the most widely used* in vivo* assays for studying anti- or proangiogenic properties [4, 12, 34]. However, theoretically, revascularization of the tumor sample is required for the sample's survival and growth [31].

The results of our study disclose that after implanting fresh LSCC tissue samples onto the chick embryo CAM the process of active angiogenesis in the CAM appears. That is the result of multiplying blood vessels associated with increase in vessel volume; hence, nascent blood vessel proliferation during our experiment is the visible sign of LSCC progression onto the CAM.

It is suggested though that alteration in the gaseous environment of chorionic epithelium may have initiated the chain of events leading to keratogenic metaplasia [46]. This phenomenon has been noticed while investigating LSCC implant onto chick embryo CAM in our study: the keratogenic metaplasia in the chorionic epithelium was observed only just beneath the implanted LSCC in contrast to the sites distant from the carcinoma implant or CAMs of control.

Results of the present study are in agreement with the data of other investigations. An increased value of vascular growth was noticed after implantation of decellularized healthy laryngeal tissue samples on CAM [24]. Ovarian fragments implanted onto chick embryo CAM markedly increased the number of distended blood vessels in the membrane near or next to the implanted ovarian fragments and an increased number of fine capillaries within close proximity of the implanted fragments were found [17, 27]. The gastrointestinal tract carcinoma cells induced angiogenesis in the CAM and positively correlated with their capacity to colonize the CAM tissue [13, 25].

The results of our study show that reliable protocol for implanting of human LSCC onto the chick embryo CAM is established and this assay can be used to analyze fresh material derived from human LSCC. However, some limitations arise from the inherent features of the chick embryo CAM model. Because the duration of the CAM assay is limited to a 7–9 days' window available before the chick hatches, most tumor cells cannot produce macroscopically visible colonies in secondary organs before the termination of the assay. As a result, the more difficult detection of micrometastases becomes an inherent part of the chick embryo CAM model system [47]. This feature of the chick embryo CAM assay probably determined a rather rare (2 cases out of 6) detection of LSCC invasion and micrometastases in our series.

## 5. Conclusions

In summary, results of our study clarify that chicken embryo CAM is a relevant host medium for implanting fresh tissues of the LSCC. The LSCC implants adhere to the host membrane and induce significant morphological changes of it, allowing visualizing microscopically the behavior of implanted tumor cells. Data of this study provide the first morphological and morphometric characterization of the LSCC implant on CAM model and, therefore, allow better understanding of cancer cell biology. Future development of this model may lead to identification of new specific and selective therapeutic agents and composition of drugs to limit spread of LSCC.

## Figures and Tables

**Table 1:** Mean thickness of the CAM: experimental group versus control group.

LSCC group			Control group		Difference			
Sight fields	Mean *μ*m	SD	Mean *μ*m	SD	*μ*m	%	*p*	*β*
1st SF	204.9	143.9	37.1	16.4	167.9	417	0.0001	<0.01^*∗*^
2nd SF	132.7	101.6			95.6	235	0.0001	<0.01^*∗*^
3rd SF	83.0	71.5			45.9	109	0.0001	<0.01^*∗*^
4th SF	124.7	98.9			87.7	214	0.0001	<0.01^*∗*^
5th SF	80.7	79.3			43.7	103	0.0001	<0.01^*∗*^

^*∗*^Statistically significant difference between the groups, computed using *α* = 0.05.

**Table 2:** Mean thickness of the CAM 2, 3, and 4 days after the LSCC tumor implanting: experimental group versus control group.

LSCC group			Control group		Difference			
Days after LSCC implantation	Mean *μ*m	SD	Mean *μ*m	SD	*μ*m	%	*p*	*β*
2nd day	102.8	97.1	37.1	16.4	66.8	177	0.001	<0.01^*∗*^
3rd day	104.9	52.8			67.9	183	0.001	<0.01^*∗*^
4th day	185.8	137.6			148.7	401	0.001	<0.01^*∗*^

^*∗*^Statistically significant difference between the groups, computed using *α* = 0.05.

**Table 3:** Mean thickness of the CAM epithelium: experimental group versus control group.

LSCC group			Control group		Difference			
Sight fields	Mean *μ*m	SD	Mean *μ*m	SD	*μ*m	%	*p*	*β*
1st SF	14.9	5.6	6.22	1.2	8.7	140	0.0001	<0.01^*∗*^
2nd SF	12.9	7.2			6.78	107	0.0001	<0.01^*∗*^
3rd SF	10.9	5.5			4.7	75	0.0001	<0.01^*∗*^
4th SF	11.9	4.7			5.7	91	0.0001	<0.01^*∗*^
5th SF	10.6	6.2			4.5	70	0.0001	<0.01^*∗*^

^*∗*^Statistically significant difference between the groups, computed using *α* = 0.05.

**Table 4:** Mean number of blood vessels per constant length of the CAM section: experimental group versus control group.

LSCC group			Control group		Difference			
Sight fields	Mean	SD	Mean	SD	Absolute	%	*p*	*β*
1st SF	15.9	10.8	6.4	2.9	9.5	148	0.0001	<0.01^*∗*^
2nd SF	12.3	5.5			5.9	92	0.0001	<0.01^*∗*^
4th SF	11.2	5.8			4.9	75	0.0001	<0.01^*∗*^

^*∗*^Statistically significant difference between the groups, computed using *α* = 0.05.

**Table 5:** Mean area of the CAM blood vessel lumen: experimental group versus control group.

LSCC group			Control group		Difference			
Sight fields	Mean *μ*m^2^	SD	Mean *μ*m^2^	SD	*μ*m^2^	%	*p*	*β*
1st SF	169.3	119.5	66.4	42.8	102.9	155	0.0001	<0.01^*∗*^
2nd SF	138.9	127.9			72.4	109	0.0001	<0.01^*∗*^
4th SF	121.0	79.7			54.6	82	0.0001	<0.05^*∗*^

^*∗*^Statistically significant difference between the groups, computed using *α* = 0.05.

## Abbreviations

LSCC: Laryngeal squamous cell carcinoma
CAM: Chorioallantoic membrane
SD: Standard deviation
SF: Sight field
HMW CK: High molecular weight cytokeratins.
